# Assessment of weaning indexes based on diaphragm activity in mechanically ventilated subjects after cardiovascular surgery. A pilot study

**DOI:** 10.5935/0103-507X.20170030

**Published:** 2017

**Authors:** Isabel Cristina Muñoz Ortega, Alher Mauricio Hernández Valdivieso, Joan Francesc Alonso Lopez, Miguel Ángel Mañanas Villanueva, Luis Horacio Atehortúa Lopez

**Affiliations:** 1 Grupo de Pesquisa em Bioinstrumentação e Engenharia Clínica, Departamento de Bioengenharia, Faculdade de Engenharia, Universidad de Antioquia - Medellín, Colômbia.; 2 Departamento de Controle Automático e Centro de Pesquisa em Engenharia Biomédica, Universitat Politécnica de Catalunya - Barcelona, Espanha.; 3 Programa de Medicina Intensiva e Crítica, Faculdade de Medicina, Universidad de Antioquia - Medellín, Colômbia.; 4 Unidade de Terapia Intensiva Cardiovascular, Hospital San Vicente Fundación - Medellín, Colômbia.

**Keywords:** Respiration, artificial, Diaphragm/physiology, Electromyography/methods, Ventilator weaning, Cardiovascular surgical procedures

## Abstract

**Objective:**

The aim of this pilot study was to evaluate the feasibility of surface electromyographic signal derived indexes for the prediction of weaning outcomes among mechanically ventilated subjects after cardiac surgery.

**Methods:**

A sample of 10 postsurgical adult subjects who received cardiovascular surgery that did not meet the criteria for early extubation were included. Surface electromyographic signals from diaphragm and ventilatory variables were recorded during the weaning process, with the moment determined by the medical staff according to their expertise. Several indexes of respiratory muscle expenditure from surface electromyography using linear and non-linear processing techniques were evaluated. Two groups were compared: successfully and unsuccessfully weaned patients.

**Results:**

The obtained indexes allow estimation of the diaphragm activity of each subject, showing a correlation between high expenditure and weaning test failure.

**Conclusion:**

Surface electromyography is becoming a promising procedure for assessing the state of mechanically ventilated patients, even in complex situations such as those that involve a patient after cardiovascular surgery.

## Abstract (pt)

**Objetivo:**

Avaliar a viabilidade do uso de índices derivados do sinal de eletromiografia de superfície para predizer desfechos do processo de desmame em pacientes mecanicamente ventilados após cirurgia cardíaca.

**Métodos:**

Foram incluídos dez pacientes em pós-operatório de cirurgia cardiovascular que não cumpriram os critérios para extubação precoce. Os sinais da eletromiografia de superfície foram registrados, assim como as variáveis ventilatórias durante o processo de desmame, sendo o momento do procedimento determinado pela equipe médica, segundo sua experiência. Avaliaram-se diversos índices da atividade dos músculos respiratórios obtidos a partir da eletromiografia de superfície com uso de técnicas de processamento lineares e não lineares. Compararam-se dois grupos: pacientes com e sem sucesso no desmame.

**Resultados:**

Os índices obtidos permitiram estimar a atividade diafragmática de cada paciente, demonstrando uma correlação entre atividade elevada e falha do teste de desmame.

**Conclusão:**

A eletromiografia de superfície está se tornando um procedimento promissor para avaliar as condições de pacientes ventilados mecanicamente, mesmo em condições complexas, como as que envolvem aqueles após cirurgia cardiovascular.

## INTRODUCTION

Despite the undeniable benefit of mechanical ventilatory assistance, its use is potentially associated with multiple complications such as damage to the airway by prolonged intubation and ventilator-associated lung injury.^(1)^

Interruption of mechanical ventilation assistance is advisable as soon as possible to protect the airway, as long as the subject is able to maintain adequate ventilation. Weaning is considered difficult when the subject meets the criteria for getting off the ventilator support and has had one or more failed attempts at extubation, or when the disconnection process cannot be completed after starting the weaning process.^(2)^

Early extubation success is associated with faster subject recovery and a significant reduction in costs associated with intensive care treatment.^(3)^ Before starting the weaning process, it is necessary that the reason leading to mechanical ventilation has been resolved satisfactorily. In addition, the subject has to be clinically and hemodynamically stable, because despite having solved the primary cause of respiratory failure, unsuccessful weaning can be influenced by several conditions, such as hemodynamic instability, acid-base disorders, electrolyte disturbances, volume overload, altered mental status, and decreased secondary myopathy muscle function.^(4)^

Repeated weaning failure has been associated with several factors, including an imbalance between the increased work of breathing (WOB) load and the reduced capacity of the diaphragm. Furthermore, it has been found that diaphragmatic dysfunction after major surgery is a common factor of respiratory failure in postcardiac surgery subjects.^(5)^

Currently, several indexes are used to predict extubation outcomes in the weaning process, such as the ratio between the respiratory rate and tidal volume (known as the rapid shallow breathing index - RSBI), the inspiratory pressure during the first 100ms, and the maximal inspiratory pressure, among others.^(6)^ However, most of them are calculated from variables related to mechanical ventilation and have not proven to be accurate enough to predict a successful weaning process.^(7-9)^

The objective of this study was to evaluate the feasibility of surface electromyographic (sEMG) signal derived indexes in the prediction of weaning outcomes among mechanically ventilated subjects after cardiac surgery. Several indexes were obtained that characterize the sEMG activity of the diaphragm in time and frequency domains. The purpose was to analyze which cases had more muscle involvement, indicating increased ventilatory work and therefore a more likely unsuccessful weaning.

## METHODS

The study was conducted according to the Helsinki's Declaration with subsequent revisions. The protocol was approved by the Ethics Committee of the *Hospital San Vicente Fundación* (Acta 01-2012, 20-01-2012), Medellín, Colombia (*Comité de* Ética *de la Investigación de Centros Especializados de San Vicente Fundación*). Written informed consent was obtained from each subject's relatives before enrollment in the study.

Considering that ventilatory mechanics takes into account a set of fluidic characteristics that allows the mobilization of gases to and from the alveoli, the clinical objective is to characterize subjects in spite of the enormous hemodynamic differences and complications of surgery. The approach proposed in this article allows assessing the respiratory system from the physical point of view using the muscular activity of the diaphragm to characterize the breathing pattern of subjects to predict the weaning outcome.

Ten male postoperative cardiac surgery subjects were enrolled in the pilot study. All of them fulfilled the inclusion criteria: adults (eighteen years and older), body mass index (BMI) less than 30, requiring mechanical ventilation after surgery and not suffering from neuromuscular disease or encephalopathy. The data collection was performed at the moment the weaning test was done by the critical care specialist based on clinical information and medical criteria. It was considered a failed weaning when the subject required either ventilatory support during 24 additional hours or reintubation past 72 hours after the weaning test. Seven subjects (63.14 ± 16.9 years old, 22.3 ± 2.5 BMI) had a successful weaning, from which 4 underwent coronary bypass surgery, 2 had a valvular replacement and 1 underwent surgical correction in annuloaortic ectasia. Three subjects (68.7 ± 7.0 years old, 25.5 ± 3.2 BMI), identified as subjects 5, 7 and 10 in the database, had a weaning failure. Of which 1 underwent a coronary bypass surgery, 1 had a valvular replacement and 1 underwent both surgeries.

### Experimental design

During the trial, sEMG signals were recorded from the diaphragm (DIA) muscle with an electromyography amplifier (Bagnoli(tm) Desktop EMG System, Delsys) with a bipolar configuration, connected to a digital acquisition card (NI USB 6212, National Instruments) with a sampling frequency of 1,024Hz. According to previous studies,^(10)^ the surface electrodes were located between the seventh and eighth intercostal space in the line, which is located in the middle of the mid axillary line and the external clavicular line. The ventilatory variables, volume, pressure, and flow, as well as the level of positive end-expiratory pressure (PEEP), lung compliance, lung resistance and respiratory rate were recorded with a Hamilton G5 mechanical ventilator with Hamilton Datalogger Software (Hamilton Medical, Bonaduz, Switzerland) with a sampling frequency of 1,024 Hz. The synchronization with ventilatory and sEMG signals was performed with a specially designed device, fully documented in.^(11)^ Table 1 describes the recorded variables and evaluated parameters with their abbreviations.

**Table 1:** Abbreviation and definitions of recorded variables and evaluated parameters

Abbreviation	Definition
Fr	Respiratory rate
PEEP	Positive end expiratory pressure level
C	Compliance
R_insp_	Inspiratory resistance
R_exp_	Expiratory resistance
Q	Flow
V	Volume
P	Pressure
DIA	Diaphragm sEMG
ML	Mean lag. Index related with the synchronization between muscular respiratory effort and ventilator response.
F_c_	Central frequency. Index related with the activation of different muscle fiber types and muscle fatigue.
Ratio HL	Ratio HL. Index related with the activation of different muscle fiber types and muscle fatigue
IM_max_	Maximal mutual information. This index offers an idea of nonlinear coupling that exists between two signals.

The traditional weaning index, RSBI, was obtained, and ventilatory mechanics of the subject were evaluated, taking into account lung compliance and the air flow resistance of the inspiratory and expiratory airway. These latter variables were obtained directly from the mechanical ventilator, which uses a least square fitting method breath by breath for estimation.^(12)^

### Signal preprocessing

**Figure 1:**
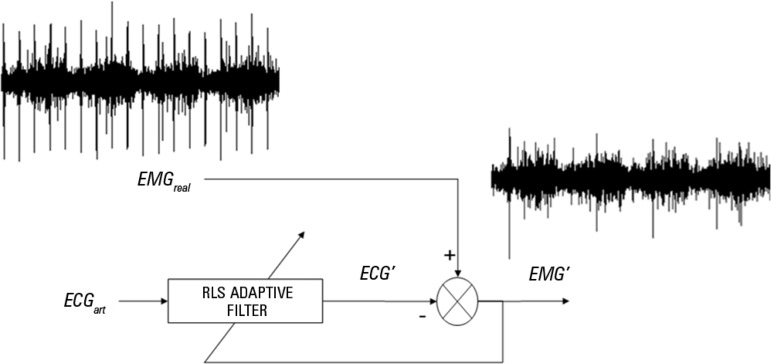
Scheme of the adaptive filter process using RLS algorithm. Where EMGreal is the recorded EMG signal that was bandpass filtered, ECGart is an artificial ECG signal obtained from EMGreal, ECG' is the ECG obtained from adaptive filter and EMG' is the EMG with a reduced cardiac interference. EMG_real_ - real electromyogram; ECG_art_ - artificial electrocardiogram; ECG - electrocardiogram.

Epochs of 180 seconds of sEMG signals were selected and a visual criterion was used to reject motion and unpredictable artifacts. A bandpass FIR filter between 10Hz and 500Hz^(13,14)^ using a Kaiser window function and a 60Hz Butterworth notch filter were used. The cardiac interference was reduced by an RLS adaptive fifth order filter.^(15)^ Figure 1 shows the main process, where EMG_real_ is the recorded EMG signal, ECG_art_ is an artificial electrocardiogram (ECG) obtained from EMG_real_ (by bandpass filtering between 5Hz and 60Hz, and then detecting the QRS complex), ECG' is the ECG signal obtained from the adaptive filter and EMG' is the desired filtered EMG signal.

### Signal processing techniques

Both linear and nonlinear signal processing techniques in time and frequency domains were used to obtain different indexes of the diaphragm's muscular activity level during mechanical ventilation.

### Power spectral density

Power spectral density (PSD) was estimated from the sEMG signals using the Burg method with order 8.^(14)^ This technique is highly recommended for these signals, because, unlike nonparametric techniques, it provides a greater frequency resolution signal in short sections like a respiratory cycle. In the Burg method, time series are modeled by an autoregressive process (AR), and PSD^(16)^ is calculated from AR model coefficients.

Two indexes were calculated from the PSD according to previous studies in subjects with obstructive apnea:

- Central or median frequency (Fc).
- Ratio between high and low frequencies (RHL): low: 20 - 40Hz and high: 138 - 240Hz.^(17)^

Both indexes permit measurement of spectral shifts during muscle contractions, which are related to the activation of different muscle fiber types and muscle fatigue.

### Cross correlation

Cross-correlation allows knowing the level of linear coupling between two time series. In this study, the relationship between muscle and ventilatory activities are important. Therefore, the correlation is calculated between the envelope of the sEMG signal and the flow signal in a respiratory cycle through equation 1:^(16)^

Equation 1 $$r_{\mathrm{xy}}\left(m\right)=\left(\frac{1}{N}\right)\sum \left(x^{2}\left(n\right)*y^{2}\left(n+m\right)\right)$$

where n is the lag or time delay, N is the number of samples and x and y represent the signals. Mean lag (ML) was defined as the mean value of the lags n with the maximum values of r_xy_ per cycle. This index is related to the synchronization among muscular effort and ventilator and respiratory responses.

### Auto mutual information

The mutual information (MI) according to information theory calculates both linear and nonlinear relations between two signals. The signals can be different, cross mutual information (CMI), or time-delayed versions of the same signal, auto mutual information (AMI), based on Shannon entropy. This technique estimates the amount of information shared by two signals, in the case of the AMI, it estimates the degree to which ξ (t+τ) can be predicted of ξ (t).^(18)^ The mutual information was calculated with equation 2:^(10)^

Equation 2 $$I\left(\xi ,\eta \right)=H\left(\eta \right)-\left[H\left(\xi ,\eta \right)-H\left(\xi \right)\right]$$

where H(η) represents the a-priori uncertainty regarding signal η, and H(ξ,η) - H(ξ)] is the remaining a-posteriori uncertainty with regard to signal η if signal ξ is known. In this pilot study, the AMI was calculated as a function of delay (τ) (AMIF) from 0 to 7 seconds.

From AMIF of the diaphragm sEMG signal, the maximum value of the main lobe (IM_max_) was estimated.

### Statistical analysis

To characterize the pattern of respiratory muscle work of the subjects, a hierarchical cluster analysis was performed taking into account all the aforementioned variables. The inconsistency coefficient (equation 3) was considered to study the dendrograms, as it provides a measure of the distance between groups related to the average nearest neighbor distance, with higher values indicating more differentiated groups.

Equation 3 $$I=\left(d-d_{m}\right)/\sigma$$

where d is the distance of the current link, d_m_ is the mean distance among links and σ is the associated standard deviation. Once the number of groups and subjects belonging to each group were identified, statistical differences between clusters were verified via the Wilcoxon-Mann-Whitney nonparametric test. Significance was set as 5%.^(19)^

## RESULTS

Table 2 summarizes the characteristics of the subjects included in the study. The anthropometric information, type of surgery and weaning outcome are specified.

**Table 2:** Characteristics of the subjects included in the study

Subject	Age (years)	BMI (kg/m^2^)	Type of surgery	Weaning outcome
1	65	24.5	Coronary bypass	Successful
2	78	20.3	Valvular replacement	Successful
3	52	16.8	Correction in annuloaortic ectasia	Successful
4	30	23	Valvular replacement	Successful
5	68	25	Coronary bypass	Unsuccessful
6	69	23.4	Coronary bypass	Successful
7	76	28.9	Coronary bypass and valvular replacement	Unsuccessful
8	72	25.5	Coronary bypass	Successful
9	76	23.4	Coronary bypass	Successful
10	62	22.5	Valvular replacement	Unsuccessful

BMI - body mass index.

**Figure 2:**
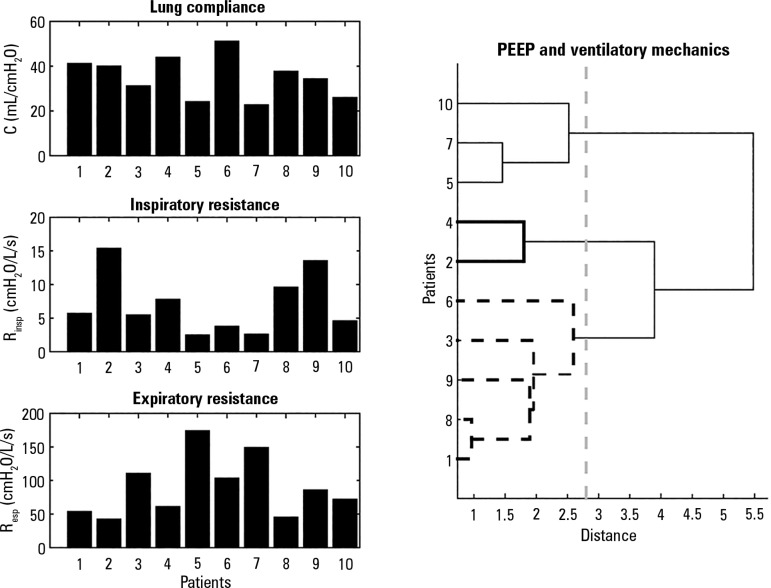
Bar diagram of ventilatory mechanics describes each case studies, on the left side from top to bottom are presented lung compliance, inspiratory and expiratory resistance measured by the Hamilton G5 ventilator by the method of least square from signals flow and pressure. In addition, on the right, classification of subjects from ventilatory mechanics and level of PEEP is presented. PEEP - positive end-expiratory pressure.

Figure 2 is a bar diagram showing values of resistance and compliance measured by the mechanical ventilator. Groups of subjects trending to high, low or medium values of those ventilatory mechanics variables can be observed. It also shows the classification obtained from a hierarchical cluster analysis including inspiratory resistance (R_insp_), expiratory resistance (R_exp_), compliance (C) and applied PEEP with the distance threshold set at 2.8, which resulted in a high inconsistency coefficient of 1.06. The group of subjects with failed weaning [5, 7, 10] showed statistically significant differences for compliance (p value = 0.05), inspiratory resistance (p value = 0.05), and PEEP (p value = 0.05), whereas expiratory resistance was not significant (p = 0.23). However, PEEP settings and breathing mechanics (C, R_insp_, R_exp_), among other mechanical ventilator parameters, are not commonly used to predict weaning outcomes. Instead, the index obtained from pressure, flow, and volume, such as RSBI is commonly used and therefore they were calculated as a reference for comparison with the indexes tested in this work. The mean and standard deviation of RSBI was 33.9 ± 14.02rpm/L, with a range [12.44 - 67.56] rpm/L.

**Figure 3:**
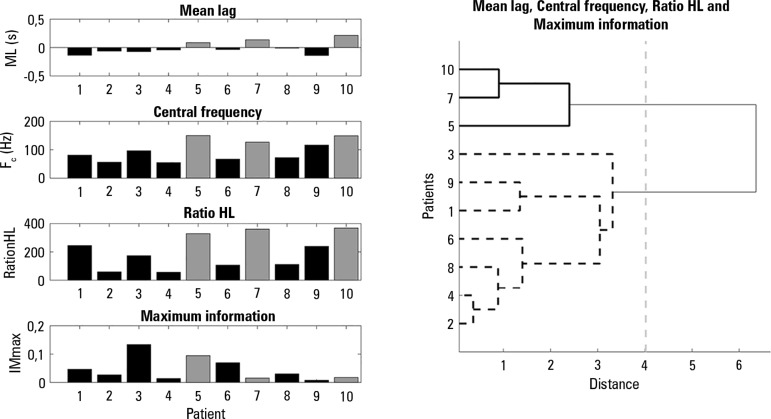
The figure on the left from top to bottom shows average of the linear and nonlinear indexes that characterize the respiratory cycle signal diaphragm muscle in 10 subjects, Mean Lag, central Frequency, Ratio HL and Maximum Mutual Information are presented. In the right plot, subject groups formed from these parameters are shown by cluster analysis with squared Euclidean distance and Ward's clustering method.

To evaluate new indexes to predict weaning outcomes, the subjects were also characterized by the level of activity of the diaphragm, which is the main muscle involved in ventilation. Figure 3 presents the proposed linear and nonlinear indexes regarding the diaphragm muscle. The dendrogram shows two clearly formed groups of subjects, with a threshold distance of 4.02, corresponding to an inconsistency coefficient of 1.13.

Interestingly, these groups correspond to the failed weaning group [5, 7, 10] and successful weaning group [1, 2, 3, 4, 6, 8, 9]. Statistical analysis revealed significant differences for the mean lag (p value = 0.005), the central frequency (p value = 0.005) and the ratio HL (p value = 0.005).

## DISCUSSION

When mechanical ventilation support is required, it must be removed in the least possible time to avoid different complications associated with mechanical ventilation. In addition, a successful extubation must be ensured because a reintubation increases complications with the clinical state of subjects.^(1)^ There are many indexes that allow prediction of the weaning outcome. The RSBI is a well-known index and the most used predictor,^(20)^ whose value, when lower than 100, has been shown to predict a successful weaning with a sensitivity of 0.97 and specificity of 0.64 in a specific population,^(21)^ although these parameters might change with different subjects and ventilator settings.^(7)^ Another study used a threshold of 65 for successful extubation of 40 postoperative surgical subjects, with a sensitivity of 0.9 and a specificity of 0.8.^(21)^ The maximal inspiratory pressure has been used to assess the inspiratory effort and has shown a low predictive value in the weaning process because of poor reproducibility between different subjects and ventilators.^(22)^ The inspiratory pressure during the first 100ms of inspiration is an indicator of central respiratory drive but its performance as a weaning predictor index depends on the strength of the respiratory muscles, meaning that results might vary between subjects with similar conditions.^(23)^ To assess the respiratory muscular strength, some indexes obtained from the neurally adjusted ventilatory assist (NAVA) catheter have been tested, but despite the good results, its performance has not been higher than that of RSBI. Additional indexes could be obtained from the NAVA catheter signal if the raw data were available to researchers.^(23)^

Different authors have assessed indexes about muscle fatigue, muscle effort and coupling of respiratory muscles from sEMG in non-ventilated subjects with chronic obstructive pulmonary disease, obstructive sleep apnea, and healthy subjects.^(2,10,17,24,25)^ However, these indexes have not been obtained in invasive ventilated subjects and therefore they have not been tested as predictors of a successful weaning process. Schmidt et al.^(26)^ found reliable results to detect the dyspnea of ventilated subjects by assessing the relationship between the intensity of dyspnea and the sEMG of intercostal and scalene muscles. This result reinforces the idea that sEMG-derived indexes could be useful to predict the weaning process outcome.

In this pilot study, several different indexes have been tested to estimate the respiratory muscle work in subjects requiring mechanical ventilation after cardiovascular surgery. Considering the hypothesis that muscle-related indexes would provide important information to decide the ideal time to wean from the mechanical ventilator, high levels of respiratory effort to maintain spontaneous breathing would indicate higher probability of failure. Otherwise, lower efforts to maintain spontaneous breathing would be related to comfortable ventilation and therefore these subjects would be candidates for successful weaning.

All subjects of this study exhibited RSBI values lower than 100 and were therefore candidates for extubation. Nevertheless, this study had two groups: the first group with 7 subjects [1, 2, 3, 4, 6, 8, 9] that had a successful weaning test and the second group with 3 subjects [5, 7, 10] that had a failed weaning test. These results are in agreement with results reported by Juern,^(21)^ who found that RSBI depends on the population under study; i.e., a new RSBI threshold of 65 in 40 postoperative surgical subjects was proposed, and we found only one subject (Subject 10) above this new threshold. In our population, it was not possible to find a threshold to discriminate the group of subjects that failed the weaning test.

Variables related to ventilatory mechanics allowed discrimination between successful and failed weaning subjects. Even in the successful group (Figure 2), there were two subjects [2, 4] that were separated from the rest of the group. Analyzing the evolution of these subjects before and after the extubation, we did not find special characteristics, so these parameters did not present high sensitivity to predict successful weaning. Along the same lines, indexes related to muscular activity of the diaphragm, such as the mean lag, the ratio HL, the central frequency and the maximum information were able to separate the subjects into the two correct groups (Figure 3).

Schmidt et al.^(26)^ and Canavan et al.^(27)^ have shown that dyspnea is highly related to asynchronies between subjects and ventilators and to a failed weaning process. Although, they found that the intensity of dyspnea was closely correlated with the EMG indexes of inspiratory muscles activity, such as intercostal and scalene, it was not measured using the diaphragm activity. In this pilot study, we tested the ML index, in which positive values indicate asynchrony with the ventilator because muscular effort during mechanical ventilator support occurs some milliseconds after breathing initiation. The failed group of subjects [5, 7, 10] had positive ML values, therefore, the relationship between asynchronies and the failed weaning process was supported and it has been proposed as a new index obtained from a noninvasive technique that assesses the diaphragm activity in mechanically ventilated subjects.

In the frequency domain, spectral indexes such as RatioHL and F_c_ from sEMG of the diaphragm showed higher values for the failed weaning group, which were related to higher efforts in the muscle during spontaneous ventilation. Parthasarathy et al.^(28)^ demonstrated that subjects that fail in the weaning process present high activity in the diaphragm, sternocleidomastoid and rib cage muscles. However, these authors captured the muscle activity with needle EMG, which is difficult to apply in an intensive care unit where noninvasive approaches are needed. We found that sEMG processed in the frequency domain could be a suitable technique to replace the invasive approach.

The automutual information (IM_max_) indicates the regularity of a signal without depending on its amplitude, therefore continuing high muscle activity might be related with high automutual information. However, in this pilot study, although the IM_max_ index along the other indexes mentioned above allowed the discrimination of subjects, individually the IM_max_ was not successful at differentiating between the failed and successful weaning groups.

As a pilot, this study had the limitation of a small sample size that only included 10 subjects, because this study is mainly focused on testing the sEMG-derived indexes and no to test efficacy of these. Additionally, it would be desirable to include a control sample in order to set thresholds to predict weaning outcomes in subjects who received cardiovascular surgery. Both limitations will be addressed in future studies including evaluation of the accessory muscles of ventilation.

## CONCLUSIONS

This pilot study suggested the utility of surface electromyography as a noninvasive diagnostic procedure for evaluating the state of mechanically ventilated subjects, even in complex situations such as those involving subjects who received cardiovascular surgery with highly compromised compliance. Due to the presented indexes being complementary, it is necessary to find a multiparametric index that relates all indexes assessed in a future work. In addition, to measure the sensitivity and specificity of proposed indexes, more subjects must be studied.
